# Impact of Sarcopenia on outcomes of pulmonary embolism: A systematic review and meta-analysis

**DOI:** 10.12669/pjms.42.5.15756

**Published:** 2026-05

**Authors:** Jie Wang, Yongmin Ding

**Affiliations:** 1 Jie Wang, Department of Pulmonary and Critical Care Medicine, Shengzhou People’s Hospital (Shengzhou Branch of the First Affiliated Hospital of Zhejiang University School of Medicine, the Shengzhou Hospital of Shaoxing University), Shengzhou, Zhejiang Province 312400, P.R. China; 2 Yongmin Ding, Department of Pulmonary and Critical Care Medicine, Shengzhou People’s Hospital (Shengzhou Branch of the First Affiliated Hospital of Zhejiang University School of Medicine, the Shengzhou Hospital of Shaoxing University), Shengzhou, Zhejiang Province 312400, P.R. China

**Keywords:** Sarcopenia, Skeletal muscle, Mortality, Pulmonary embolism

## Abstract

**Background and Objective:**

Sarcopenia measured by, skeletal muscle mass, has emerged a marker of adverse outcomes in various acute illnesses. Its prognostic significance in patients with acute pulmonary embolism (PE) remains incompletely defined. This systematic review and meta-analysis aimed to evaluate the association between sarcopenia and clinical outcomes in acute PE.

**Methodology:**

A systematic search of PubMed, Embase, Web of Science, and Scopus was conducted from inception to December 27, 2025. Observational studies evaluating sarcopenia using computed tomography (CT)-based skeletal muscle parameters in adults with acute PE and reporting short-term mortality (mortality within 90 days) or in-hospital outcomes were included. Random-effects meta-analysis was performed when data were amenable to pooling.

**Results:**

Six observational studies comprising 578,886 patients met the inclusion criteria. A pooled analysis of four studies demonstrated that higher skeletal muscle mass or index was associated with a reduced risk of short-term mortality (odds ratio: 0.94; 95% confidence interval: 0.90–0.98), although substantial heterogeneity was observed (I² = 75%) and results were unstable on sensitivity analyses. Descriptive analysis of two studies showed worse in-hospital outcomes in patients with sarcopenia.

**Conclusions:**

The assessment of sarcopenia through CT-derived skeletal muscle measurements may be associated with an increased risk of short-term mortality among patients with acute PE. Quantitative analyses of data also showed a correlation between sarcopenia and adverse outcomes. Methodological heterogeneity and scarce data are important limitations of the review.

***Registration No:*** PROSPERO (CRD420251273131).

## INTRODUCTION

Acute pulmonary embolism (PE) is a potentially fatal cardiovascular emergency which can present in a variety of ways ranging from asymptomatic cases to instances of hemodynamic instability and sudden mortality.^1^ Despite significant advancements in diagnostic imaging modalities and therapeutic interventions, the incidence of short-term mortality remains considerable, particularly among the elderly and individuals with diminished physiological reserves.^2^ Therefore, precise risk stratification at the initial clinical assessment is important to guide management strategies, including the necessity for intensive monitoring or the implementation of advanced reperfusion procedures.^3^ Presently, prognostic evaluation in acute PE primarily is based on clinical parameters, biomarker analysis, and imaging markers of right ventricular dysfunction.^3^,^4^ Although these methodologies offer valuable insights, they may inadequately reflect the patient’s intrinsic vulnerability. Increasingly, frailty and diminished physiological reserve have been recognised as critical determinants influencing clinical outcomes in acute illnesses, especially within geriatric populations.^5^ Sarcopenia, defined by the loss of skeletal muscle mass and decline in muscle quality, emerges as a significant biological component of frailty, consistently linked to poorer prognoses across various diseases.^6^,^7^

In routine clinical practice, skeletal muscle mass assessment is often performed using cross-sectional imaging, with computed tomography (CT) as the most common modality. Quantitative analysis of muscle tissue obtained from CT scans has demonstrated utility as a pragmatic surrogate marker for sarcopenia, especially in acutely ill patients where functional assessments are impractical.^8^ Typically, muscle mass evaluation has targeted measurements at the lumbar third vertebral (L3) level; however, this region is not routinely included in CT pulmonary angiography (CTPA), the standard diagnostic procedure for PE.

Recent research has thus investigated alternative thoracic CT-derived muscle parameters available from CTPA images. These include measurements of muscles such as the psoas, paraspinal, and pectoralis at multiple thoracic levels.^9^ Preliminary findings suggest that diminished thoracic skeletal muscle mass and compromised muscle quality may be linked to increased short-term mortality and hemodynamic instability among patients with acute PE.^9^,^10^ Despite these promising insights, current data are limited in scope, exhibit methodological heterogeneity, and are predominantly based on small or single-center cohorts.

Given the rising interest in body composition as a prognostic biomarker and the increasing use of CT-based muscle assessment in routine PE imaging, a comprehensive review of current evidence is necessary. This systematic review and meta-analysis aimed to assess how sarcopenia affects clinical outcomes in patients with acute PE, focusing primarily on short-term mortality.

## METHODOLOGY

This systematic review and meta-analysis were conducted according to the Preferred Reporting Items for Systematic Reviews and Meta-Analyses (PRISMA) 2020 guidelines.^11^ It was registered on PROSPERO (CRD420251273131).

### Search strategy:

A thorough search of the literature was conducted across PubMed, Embase, Web of Science, and Scopus from their inception until December 27, 2025. The search protocols incorporated both controlled vocabulary and free-text keywords without imposing any language restrictions. The comprehensive electronic search strategies for each database are detailed in Supplementary Table-I. Additionally, reference lists of included articles and pertinent reviews were manually examined to identify further eligible studies. The literature search was performed independently by two reviewers, with discrepancies resolved through discussion.

**Supplementary Table-I:** Search Strategy.

***1. PubMed (MEDLINE)***
((“Pulmonary Embolism”[Mesh] OR “pulmonary embolism”[Title/Abstract] OR “acute pulmonary embolism”[Title/Abstract] OR PE[Title/Abstract]) AND (“Sarcopenia”[Mesh] OR sarcopenia[Title/Abstract] OR “skeletal muscle”[Title/Abstract] OR “muscle mass”[Title/Abstract] OR “muscle area”[Title/Abstract] OR “muscle index”[Title/Abstract] OR psoas[Title/Abstract] OR pectoralis[Title/Abstract]))
***2. Embase***
(‘pulmonary embolism’/exp OR ‘pulmonary embolism’:ti,ab OR ‘acute pulmonary embolism’:ti,ab OR PE:ti,ab) AND (‘sarcopenia’/exp OR sarcopenia:ti,ab OR ‘skeletal muscle’:ti,ab OR ‘muscle mass’:ti,ab OR ‘muscle area’:ti,ab OR ‘muscle index’:ti,ab OR psoas:ti,ab OR pectoralis:ti,ab)
***3. Scopus***
(TITLE-ABS-KEY(“pulmonary embolism” OR “acute pulmonary embolism” OR PE) AND TITLE-ABS-KEY(sarcopenia OR “skeletal muscle” OR “muscle mass” OR “muscle area” OR “muscle index” OR psoas OR pectoralis))
***4. Web of Science***
(TS=(“pulmonary embolism” OR “acute pulmonary embolism” OR PE) AND TS=(sarcopenia OR “skeletal muscle” OR “muscle mass” OR “muscle area” OR “muscle index” OR psoas OR pectoralis))

### Eligibility criteria:

Studies were considered eligible if they met the following criteria:

- Included adult patients (≥18 years) with acute PE.
- Assessed sarcopenia using skeletal muscle parameters using CT-based measurements (e.g., muscle area, thickness, density, skeletal muscle index) or diagnostic coding.
- Reported at least one clinical outcome, either short-term mortality or in-hospital adverse events.
- Used an observational study design (cohort or registry-based studies).

Studies were excluded if they did not specifically evaluate PE or skeletal muscle–related parameters, and were review articles, case reports, editorials, or 3. were conference abstracts without complete texts.

### Study selection:

All citations were organized and deduplicated using EndNote (version 21). Two independent reviewers screened all search results through a three-step process:

- Removing duplicates.
- Screening titles and abstracts.
- Reviewing full texts for eligibility.
- Studies deemed relevant by either reviewer were downloaded for full-text assessment. Any disagreements were resolved through discussion.

### Data extraction:

Data were independently extracted using a standardized form. For each included study, the following details were recorded: author and publication year, country, study design, sample size, patient demographics (age and sex), reported comorbidities, methods used to assess sarcopenia, reported sarcopenia prevalence, and relevant outcomes. The primary outcome of interest was short-term mortality, defined as mortality occurring during hospitalization or within 90 days of diagnosis. Other adverse events were considered as secondary outcomes.

### Risk of bias assessment:

The methodological quality of the included observational studies was assessed using the Newcastle–Ottawa Scale (NOS).^12^ This evaluation focused on three areas: selection of study groups, group comparability, and outcome ascertainment. Studies could earn up to nine points. Those scoring seven or higher were deemed low risk; scores between four and six indicated moderate risk; and scores of three or less indicated a high risk of bias. Two reviewers conducted the risk of bias analysis and resolved differences by discussion.

### Statistical analysis:

Quantitative synthesis was conducted only when at least two studies provided appropriate data suitable for meta-analytic procedures. We extracted adjusted odds ratios (OR) data from the studies and derived pooled ORs and their corresponding 95% confidence intervals (CIs) utilizing a random-effects model in Review Manager (RevMan version 5.3) software. No data conversion was needed. The statistical heterogeneity was evaluated via the I² statistic, with values exceeding 50% indicating substantial heterogeneity. Sensitivity analyses involved sequentially omitting individual studies to assess the stability of the pooled estimates. Due to the limited number of included studies, publication bias was not assessed.

## RESULTS

### Search results and baseline details:

**Fig.1:**
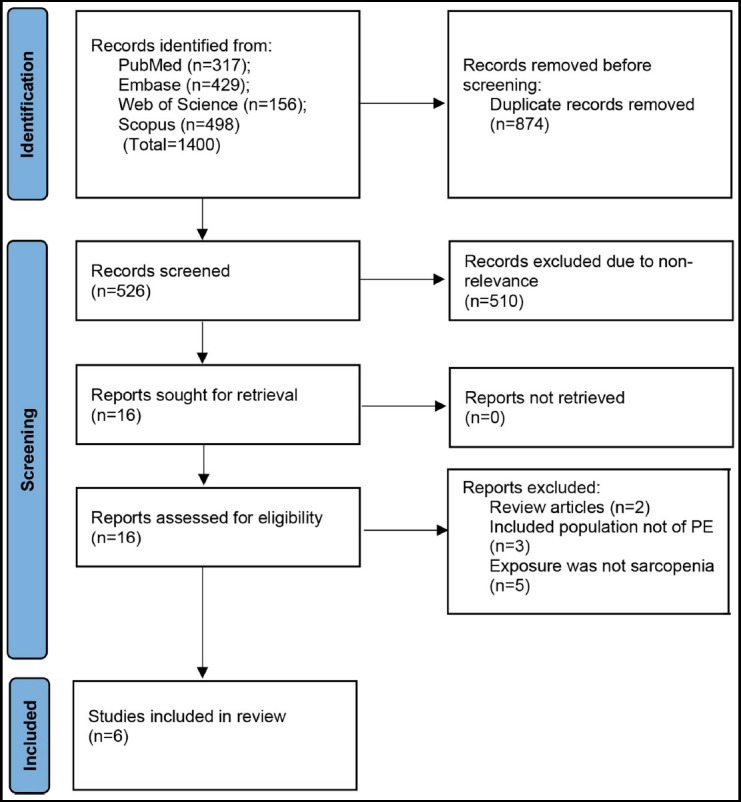
Study flowchart.

The search yielded 1,400 records. After removing 874 duplicate entries, 526 unique records were screened based on titles and abstracts. Of these, 510 records were excluded because they did not meet the research criteria. Full texts of 16 articles were subsequently retrieved and evaluated for eligibility; of these, 10 were excluded based on predefined exclusion criteria. Ultimately, six studies satisfied the inclusion parameters (Fig.1).^4^,^9^,^10^, ^3^-^15^

The six included studies comprised a total of 578,886 patients with acute PE (Table-I). Four studies were conducted in Germany, and two in Turkey. Sample sizes varied markedly, ranging from 89 to 576,364 patients. The mean or median age across studies ranged from 63 to 82 years, and the proportion of male participants ranged from 35.7% to 57.3%. Reporting of baseline comorbidities was heterogeneous. D-dimer levels were reported in only three studies. Assessment of sarcopenia and skeletal muscle parameters differed considerably across studies. Muscle evaluation was performed using thoracic CT-based measurements in four studies, including pectoralis muscle thickness or area and thoracic skeletal muscle indices at levels T4-T5. One study assessed lumbar psoas muscle area at the L3 level, normalized to body surface area, while one extensive administrative study identified sarcopenia using the International Classification of Diseases (ICD-10) diagnostic coding. Only one study reported the prevalence of sarcopenia, which was 0.4% in an elderly inpatient cohort. The risk of bias analysis of the included studies is shown in Table-II. According to NOS, three studies scored eight points, while three scored six points.

**Table-I:** Details of included studies.

Study Location	Sample size	Age (Years)	Male (%)	DM (%)	HT (%)	Prior DVT (%)	D-Dimer	Sarcopenia assessment	Variables adjusted	Sarcopenia prevalence (%)	Outcomes
Tasci 2024 Turkey	389	73 (63-82)	35.7	38.6	81.2	1.5	3,200 (1,576–5,670) µg/L	Sarcopenia assessed indirectly via bilateral pectoralis muscle thickness measured at the T5 vertebral level on CT axial images. Measurements were performed using AW4.6 software, with standardized anatomical landmarks and a single experienced radiologist to ensure consistency.	NR	NR	90-day mortality
Keller 2024 Germany	576364	82	36.7	22.9	52.2	32.3	NR	ICD-10 code for sarcopenia	Age, sex, obesity, hyperlipidaemia, cancer, coronary artery disease, heart failure, chronic obstructive pulmonary disease, HT, kidney failure, DM, and atrial fibrillation/flutter	0.4	In-hospital mortality; adverse events; Length of stay
Shahzadi 2024 Germany	829	65 (18–100)	53.6	NR	NR	NR	NR	Skeletal muscle (SM) and intramuscular adipose tissue (IMAT) were segmented on a single axial slice at the level of thoracic vertebra T12. Segmentation was performed using ImageJ software with predefined attenuation thresholds (SM: −29 to +150 HU; IMAT: −190 to −30 HU). A total of 234 radiomic features (morphological, first-order, texture, and LoG features) were extracted using the MIRP radiomics platform, following IBSI standards.	Age, sex, PESI	NR	7-day and 30-day mortality
Meyer 2022 Germany	234	63.9 ± 16.7	53	NR	NR	NR	17.6 ± 24.6 ng/mL	Pectoralis muscle measurements were performed on CT axial images at the T4 level, including both pectoralis major and minor muscles. Skeletal Muscle Index was derived by normalising Skeletal Muscle Area to height (cm²/m²). Measurements were performed using dedicated PACS software by blinded radiologists.	Age, sex, body mass index, and PESI	NR	30-day mortality
Meyer 2023 Germany	981	63.5 ± 15.9	55.1	NR	NR	NR	NR	Skeletal muscle area was quantified at the thoracic vertebra T5 (T5) level using semi-automated segmentation (ImageJ software) with predefined attenuation thresholds (–29 to +150 HU) on CT. Skeletal Muscle Index was calculated by normalizing muscle area to height	Age, sex, systolic blood pressure, PESI, and presence of active malignancy	NR	30-day mortality
Akkoc 2020 Turkey	89	67.5 ± 15.4	57.3	NR	NR	NR	8,254 ± 2,078 ng/mL	CT-based measurement performed on abdominal scans at the caudal end of the L3 vertebra. Bilateral psoas muscle cross-sectional areas were measured by a blinded radiologist, averaged over three readings, and normalized to body surface area (BSA).	PESI, heart rate, respiratory rate, systolic blood pressure, and arterial oxygen saturation	NR	In-hospital mortality

Diabetes mellitus; DVT, deep vein thrombosis; NR, not reported; HT, hypertension; PE, pulmonary embolism; T, thoracic; L, lumbar; BSA, body surface area; CT, computed tomography; ICD, international classification of diseases; PESI, Pulmonary embolism severity index

**Table-II:** Risk of bias analysis of included studies.

Study	Selection of studies	Comparability	Outcomes	Total Score
Tasci 2024	4	-	2	6
Keller 2024	4	-	2	6
Shahzadi 2024	4	-	2	6
Meyer 2022	4	2	2	8
Meyer 2023	4	2	2	8
Akkoc 2020	4	2	2	8

### Meta-analysis:

**Fig.2:**
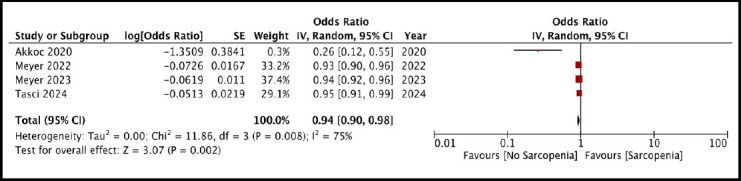
Meta-analysis of the association between sarcopenia and mortality after PE.

Data for the meta-analysis were available from four studies. Pooled analysis showed that higher skeletal muscle mass/index was associated with reduced risk of short-term mortality in PE patients (OR: 0.94, 95% CI: 0.90, 0.98, I^2^=75%) (Fig.2). The results were unstable in the sensitivity analysis and became non-significant after excluding the studies of Meyer et al.^10^ Two studies, including the large nationwide study by Keller et al.^14^, did not present data amenable to meta-analysis due to differences in sarcopenia definition (ICD-based coding) and outcome reporting; hence, their findings were analyzed descriptively.

### Descriptive analysis:

In a comprehensive nationwide retrospective study encompassing 576,364 hospitalized patients aged 75 years and older with a diagnosis of acute PE, Keller et al.^14^ examined the presence of sarcopenia as identified through ICD-10 diagnostic coding. Patients classified as sarcopenic were characterized by advanced age and a markedly greater burden of comorbidities and frailty-associated conditions compared to their non-sarcopenic counterparts. Clinically, the presence of sarcopenia correlated with a more severe in-hospital clinical trajectory, evidenced by increased incidences of shock, tachycardia, pneumonia, acute kidney injury, stroke, gastrointestinal bleeding, and the requirement for blood transfusions. Additionally, sarcopenic patients experienced a significantly prolonged hospitalization duration. Notwithstanding this elevated risk profile, the utilisation of systemic thrombolytic or reperfusion therapies was less frequent among sarcopenic patients relative to non-sarcopenic patients. These associations remained statistically significant after adjustment for multiple confounders.

Shahzadi et al.^15^ conducted a multicenter retrospective investigation involving 829 patients diagnosed with acute PE. The study utilized radiomics-based analysis of skeletal muscle and intramuscular adipose tissue (IMAT) derived from CTPA at the T12 vertebral level. Rather than categorizing sarcopenia dichotomously, muscle characteristics were evaluated through high-dimensional radiomic features. The primary endpoints consisted of all-cause mortality at seven and 30 days. Radiomic models utilizing IMAT alone and in combination with skeletal muscle demonstrated moderate predictive capacity for 30-day mortality, with test-set area under the receiver operating characteristic curves ranging from approximately 0.68 to 0.70. Conversely, radiomic signatures showed limited predictive value for seven-day mortality. Overall, the findings indicate that radiomics-assessed muscle quality and adipose infiltration are associated with short-term mortality risk in patients with acute PE.

## DISCUSSION

This systematic review and meta-analysis are the first in the literature to examine the association between sarcopenia and prognosis in patients with acute PE. The analysis showed that greater skeletal muscle mass or index is associated with reduced short-term mortality risk, though there was significant heterogeneity. Moreover, the results were unstable during sensitivity analysis. The quantitative synthesis of two large studies that couldn’t be included in the meta-analysis offered additional supporting evidence indicating worse outcomes with sarcopenia.

Sarcopenia is regarded as a hallmark of ageing and is characterized by a gradual and widespread loss of skeletal muscle mass, strength, and function.^16^ The worldwide prevalence of sarcopenia is about 10-27% based on various definitions and cut-offs.^17^ Multiple factors have been implicated in the development of sarcopenia, including decreased physical activity, poor nutrition, systemic inflammation, and chronic illnesses.^18^ Importantly, sarcopenia is now being increasingly recognised as an independent risk factor for adverse outcomes after several diseases, including cardiovascular diseases^6^,^7^ and malignancies.^19^ A meta-analysis by Li et al.,^20^ including nine datasets has shown that premorbid sarcopenia led to worse functional outcomes in patients with stroke. Another study by Pan et al.^21^ has shown that patients with sarcopenia, assessed by a surrogate index, had high all-cause mortality after myocardial infarction.

However, there remains scarce evidence on the effect of sarcopenia on outcomes of acute PE. The evidence from our study complements published evidence suggesting that sarcopenia may also be a risk factor for short-term mortality in acute PE patients; however, the data are scarce and not robust to sensitivity analysis, and should therefore be interpreted with caution. Only one other study^14^ examined the association between sarcopenia and other adverse outcomes and noted that in-hospital complications were increased with the presence of sarcopenia along with reduced utilisation of reperfusion therapies. Similar results have been reported by Schmitt et al.,^22^ wherein sarcopenia was associated with decreased utilisation of endovascular reperfusion in patients with peripheral artery disease.

The variability in definitions of sarcopenia has posed a considerable challenge in the existing literature.^23^ Multiple research groups have proposed operational criteria for diagnosing the condition, emphasizing outcomes such as reduced muscle mass, functional decline, and muscle weakness; some also consider grip strength and gait velocity as key indicators.^24^ Despite ongoing efforts, a universally accepted definition has yet to be established, though consensus-building is ongoing.^25^ Initially characterized primarily by loss of muscle mass, recent perspectives have shifted towards emphasizing a decline in skeletal muscle function, particularly strength. The optimal method for assessing these parameters remains uncertain.^24^ The Global Leadership Initiative on Sarcopenia (GLIS) was recently founded to develop a comprehensive, universally endorsed definition, supported by leading consensus groups, including the Australian and New Zealand Society for Sarcopenia and Frailty Research and the European Working Group on Sarcopenia in Older People. Nevertheless, a significant obstacle persists in the inconsistent reliability of measurement techniques for muscle mass, necessitating further research.^25^ This issue was also evident in the current review, where studies used varying definitions and measurement approaches for sarcopenia (pectoralis muscle thickness, skeletal muscle index at T4–T5, psoas muscle area at L3, radiomics analysis, and ICD coding). Some studies used skeletal muscle mass while others used skeletal muscle index. We believe that this was one of the primary reasons for the high heterogeneity in the meta-analysis. These approaches can’t be directly compared because they involve different body regions, measurement methods, and aspects of muscle composition, including both quantity and quality. To reduce methodological inconsistency, only studies with similar CT-based muscle measurements were included in the overall analysis. Studies that used very different definitions were described separately. However, even among the pooled studies, there was still variation in the specific anatomical points used, normalization techniques, and cutoff values. Because of this, the overall estimates should be viewed with caution. A detailed subgroup analysis was not feasible due to limited data in the literature; further studies are needed to identify the optimal definition of sarcopenia, especially in PE patients.

Sarcopenia may elevate the risk of mortality in patients with acute PE through various interconnected pathophysiological mechanisms. These mechanisms reflect diminished physiological reserve, impaired cardiopulmonary adaptability, and adverse metabolic signaling pathways. The loss of skeletal muscle mass and decline in muscle quality are indicative of frailty and are linked to reduced capacity to withstand acute hemodynamic challenges, hypoxemia, and inflammatory insults.^26^ In the context of acute PE, characterized by right ventricular pressure overload and sudden increases in pulmonary vascular resistance, sarcopenia may indicate an underlying inability to mount effective compensatory responses.^10^ Recent evidence suggests that skeletal muscle functions as an endocrine organ, secreting cardioprotective myokines such as irisin, musclin, and myonectin, which exhibit anti-apoptotic, anti-inflammatory, and pro-angiogenic effects on the myocardium and pulmonary vasculature. A reduction in muscle mass and the presence of increased intramuscular adipose tissue, may correlate with decreased secretion of these myokines, thereby promoting myocardial vulnerability, hindering right ventricular adaptation, and increasing susceptibility to ischemia–reperfusion injury.^21^ Additionally, heightened intramuscular fat infiltration signifies metabolic dysregulation and chronic low-grade inflammation, factors that can exacerbate endothelial dysfunction and compromise microcirculatory reserve during acute PE.^15^

### Limitations:

The NOS score of all the studies was not high which limits the quality of the evidence. Moreover, it’s important to consider several key sources of bias when analyzing the findings of this review. Most included studies were retrospective, which inherently introduces selection bias and limits the ability to establish causality. Residual confounding remains a concern, as sarcopenia is associated with factors like frailty, aging, malnutrition, and increased comorbidities. Many studies did not fully control for these variables, meaning that some observed associations might reflect patient vulnerability rather than a direct impact of sarcopenia. Additionally, important baseline variables and comorbidities were inconsistently reported, complicating subgroup analyses. Variations in CT protocols and muscle measurement techniques, such as differences in anatomical landmarks, segmentation methods, and normalization, also contributed to measurement bias and hindered comparability across studies. Two studies could not be included in the quantitative analysis because their definitions of exposure and reporting of outcomes were too different; only a descriptive summary was possible. Notably, one of the studies of Keller et al.^14^ comprised a very large nationwide cohort, which, although not included in the pooled analysis, may have disproportionately affected the interpretation of the results. The dependence on ICD coding to define sarcopenia in this study further introduces the possibility of misclassification bias. Further, the definition of short-term mortality varied across studies and included in-hospital, 30-day, and 90-day mortality, which may have introduced minor inconsistencies in outcome assessment and contributed to heterogeneity. Lastly, the small number of available studies limited the ability to properly assess publication bias and explore sources of heterogeneity.

### Clinical implications.

This review indicates that skeletal muscle assessment on CT may provide additional prognostic information in patients with acute PE. This approach can complement existing clinical risk scores. Utilizing opportunistic evaluation of skeletal muscle parameters from routinely performed CTPA may facilitate the identification of patients with diminished physiological reserve, who could potentially benefit from intensified monitoring or tailored therapeutic interventions. Prior to integrating such assessments into routine clinical practice, prospective, multicenter studies are required to establish standardized thoracic muscle measurement protocols, determine clinically relevant cutoff values, and assess their incremental value beyond current risk stratification methods. Future research should also focus on developing automated, radiomics-based muscle assessment techniques suitable for clinical workflows, as well as on investigating whether interventions to counteract sarcopenia can enhance patient outcomes in the context of acute PE.

## CONCLUSIONS

The assessment of sarcopenia through CT-derived skeletal muscle measurements may be associated with an increased risk of short-term mortality among patients with acute PE. Quantitative analyses of data also showed a correlation between sarcopenia and adverse outcomes. Methodological heterogeneity and scarce data are important limitations of the review.

### Author’s Contributions:

**JW:** Literature search, study design and manuscript writing. revision and validation and are responsible for the integrity of the study.

**JW and YD:** Data collection, data analysis and interpretation. Critical review.

All authors have read and approved the final manuscript.
